# No Association of Coronary Artery Disease with X-Chromosomal Variants in Comprehensive International Meta-Analysis

**DOI:** 10.1038/srep35278

**Published:** 2016-10-12

**Authors:** Christina Loley, Maris Alver, Themistocles L. Assimes, Andrew Bjonnes, Anuj Goel, Stefan Gustafsson, Jussi Hernesniemi, Jemma C. Hopewell, Stavroula Kanoni, Marcus E. Kleber, King Wai Lau, Yingchang Lu, Leo-Pekka Lyytikäinen, Christopher P. Nelson, Majid Nikpay, Liming Qu, Elias Salfati, Markus Scholz, Taru Tukiainen, Christina Willenborg, Hong-Hee Won, Lingyao Zeng, Weihua Zhang, Sonia S. Anand, Frank Beutner, Erwin P. Bottinger, Robert Clarke, George Dedoussis, Ron Do, Tõnu Esko, Markku Eskola, Martin Farrall, Dominique Gauguier, Vilmantas Giedraitis, Christopher B. Granger, Alistair S. Hall, Anders Hamsten, Stanley L. Hazen, Jie Huang, Mika Kähönen, Theodosios Kyriakou, Reijo Laaksonen, Lars Lind, Cecilia Lindgren, Patrik K. E. Magnusson, Eirini Marouli, Evelin Mihailov, Andrew P. Morris, Kjell Nikus, Nancy Pedersen, Loukianos Rallidis, Veikko Salomaa, Svati H. Shah, Alexandre F. R. Stewart, John R. Thompson, Pierre A. Zalloua, John C. Chambers, Rory Collins, Erik Ingelsson, Carlos Iribarren, Pekka J. Karhunen, Jaspal S. Kooner, Terho Lehtimäki, Ruth J. F. Loos, Winfried März, Ruth McPherson, Andres Metspalu, Muredach P. Reilly, Samuli Ripatti, Dharambir K. Sanghera, Joachim Thiery, Hugh Watkins, Panos Deloukas, Sekar Kathiresan, Nilesh J. Samani, Heribert Schunkert, Jeanette Erdmann, Inke R. König

**Affiliations:** 1 Institut für Medizinische Biometrie und Statistik, Universität zu Lübeck, Universitätsklinikum Schleswig-Holstein, Campus Lübeck, Lübeck, Germany; 2 DZHK (German Centre for Cardiovascular Research), partner site Hamburg–Lübeck–Kiel, Lübeck, Germany; 3 Estonian Genome Center, University of Tartu, Tartu, Estonia; 4 Institute of Molecular and Cell Biology, Tartu, Estonia; 5 Department of Medicine, Division of Cardiovascular Medicine, Stanford University School of Medicine Stanford, Standford, California, USA; 6 Center for Human Genetic Research, Massachusetts General Hospital, Boston, Massachusetts, USA; 7 Division of Cardiovascular Medicine, Radcliffe Department of Medicine, University of Oxford, Oxford, UK; 8 Wellcome Trust Centre for Human Genetics, University of Oxford, Oxford, UK; 9 Department of Medical Sciences, Molecular Epidemiology and Science for Life Laboratory, Uppsala University, Uppsala, Sweden; 10 Department of Clinical Chemistry, Fimlab Laboratories, Tampere, Finland; 11 Department of Cardiology, Heart Hospital and University of Tampere School of Medicine, Tampere, Finland; 12 CTSU, Nuffield Department of Population Health, University of Oxford, Oxford, UK; 13 William Harvey Research Institute, Barts and The London School of Medicine and Dentistry, Queen Mary University of London, London, UK; 14 Vth Department of Medicine, Medical Faculty Mannheim, Heidelberg University, Mannheim, Germany; 15 The Charles Bronfman Institute for Personalized Medicine, Icahn School of Medicine at Mount Sinai, New York, USA; 16 Department of Clinical Chemistry, University of Tampere School of Medicine, Tampere, Finland; 17 Department of Cardiovascular Sciences, University of Leicester, Leicester, UK; 18 NIHR Leicester Cardiovascular Biomedical Research Unit, Glenfield Hospital, Leicester, UK; 19 Ruddy Canadian Cardiovascular Genetics Centre University of Ottawa Heart Institute, Ottawa, Canada; 20 Department of Biostatistics and Epidemiology, University of Pennsylvania, Philadelphia, Pennsylvania, USA; 21 Institute for Medical Informatics, Statistics and Epidemiology/Medical Faculty/University of Leipzig, Leipzig, Germany; 22 LIFE Research Center of Civilization Diseases, Leipzig, Germany; 23 Analytic and Translation Genetics Unit, Massachusetts General Hospital, Boston, Massachusetts, USA; 24 Program in Medical and Population Genetics, Broad Institute, Cambridge, Massachusetts, USA; 25 Institut für Integrative und Experimentelle Genomik, Universität zu Lübeck, Universitätsklinikum Schleswig-Holstein, Campus Lübeck, Lübeck, Germany and University Heart Center Luebeck, Campus Lübeck, Lübeck, Germany; 26 Samsung Advanced Institute for Health Sciences and Technology (SAIHST), Sungkyunkwan University, Samsung Medical Center, Seoul, Korea; 27 Deutsches Herzzentrum München, Technische Universität München, Munich, Germany; 28 DZHK (German Centre for Cardiovascular Research), partner site Munich Heart Alliance, München, Germany; 29 Department of Epidemiology and Biostatistics, Imperial College London, London, UK; 30 Department of Cardiology, Ealing Hospital National Health Service (NHS) Trust, Middlesex, UK; 31 Population Health Research Institute, McMaster University, Hamilton, Ontario, Canada; 32 Heart Center Leipzig, Cardiology, University of Leipzig, Leipzig, Germany; 33 Harokopio University Athens, Athens, Greece; 34 The Center for Statistical Genetics, Icahn School of Medicine at Mount Sinai, New York, USA; 35 The Icahn Institute for Genomics and Multiscale Biology, Icahn School of Medicine at Mount Sinai, New York, USA; 36 The Zena and Michael A. Weiner Cardiovascular Institute, Icahn School of Medicine at Mount Sinai, New York, USA; 37 Department of Medicine, Harvard Medical School, Boston, Massachusetts, USA; 38 INSERM, UMRS1138, Centre de Recherche des Cordeliers, Paris, France; 39 Department of Public Health and Caring Sciences, Geriatrics, Uppsala Universit, Uppsala, Sweden; 40 Duke University School of Medicine, Durham, North Carolina, USA; 41 Leeds Institute of Genetics, Health and Therapeutics, University of Leeds, UK; 42 Cardiovascular Genetics and Genomics Group, Atherosclerosis Research Unit, Department of Medicine Solna, Karolinska Institutet, Stockholm, Sweden; 43 Cleveland Clinic, Cleveland, Ohio, USA; 44 Boston VA Research Institute, Inc., Boston, Massachusetts, USA; 45 Department of Clinical Physiology, Tampere University Hospital, Tampere, Finland; 46 Department of Clinical Physiology, University of Tampere School of Medicine, Tampere, Finland; 47 Zora Biosciences, Espoo, Finland; 48 Department of Medical Sciences, Cardiovascular Epidemiology, Uppsala University, Uppsala, Sweden; 49 Broad Institute of the Massachusetts Institute of Technology and Harvard University, Cambridge, Massachusetts, USA; 50 Department of Medical Epidemiology and Biostatistics, Karolinska Institutet, Stockholm, Sweden; 51 Department of Biostatistics, University of Liverpool, Liverpool, UK; 52 Second Department of Cardiology, University General Hospital Attikon, Athens, Greece; 53 Department of Chronic Disease Prevention, National Institute for Health and Welfare, Helsinki, Finland; 54 Department of Health Sciences, University of Leicester, Leicester, UK; 55 Lebanese American University, School of Medicine, Beirut, Lebanon; 56 Harvard School of Public Health, Boston, Massachusetts, USA; 57 Imperial College Healthcare NHS Trust, London, UK; 58 Kaiser Permanente, Division of Research, Oakland, California, USA; 59 Department of Forensic Medicine, University of Tampere School of Medicine, Tampere, Finland; 60 Cardiovascular Science, National Heart and Lung Institute, Imperial College London, London, UK; 61 The Mindich Child Health and Development Institute, Icahn School of Medicine at Mount Sinai, New York, USA; 62 Synlab Academy, Synlab Services GmbH, Mannheim, Germany; 63 Clinical Institute of Medical and Chemical Laboratory Diagnostics, Medical University of Graz, Graz, Austria; 64 Cardiovascular Institute, Perelman School of Medicine at the University of Pennsylvania, Philadelphia, Pennsylvania, USA; 65 Hjelt Institute, University of Helsinki, Helsinki, Finland; 66 Institute for Molecular Medicine Finland FIMM, University of Helsinki, Helsinki, Finland; 67 Department of Pediatrics, College of Medicine, University of Oklahoma Health Sciences Center, Oklahoma City, Oklahoma, USA; 68 Department of Pharmaceutical Sciences, College of Pharmacy, University of Oklahoma Health Sciences Center, Oklahoma City, Oklahoma, USA; 69 Oklahoma Center for Neuroscience, Oklahoma City, Oklahoma, USA; 70 Institute for Laboratory Medicine, Clinical Chemistry and Molecular Diagnostics, University Hospital Leipzig, Medical Faculty, Leipzig, Germany; 71 Wellcome Trust Sanger Institute, Hinxton, Cambridge, UK; 72 Princess Al-Jawhara Al-Brahim Centre of Excellence in Research of Hereditary Disorders (PACER-HD), King Abdulaziz University, Jeddah, Saudi Arabia; 73 Cardiovascular Research Center, Massachusetts General Hospital, Boston, Massachusetts, USA

## Abstract

In recent years, genome-wide association studies have identified 58 independent risk loci for coronary artery disease (CAD) on the autosome. However, due to the sex-specific data structure of the X chromosome, it has been excluded from most of these analyses. While females have 2 copies of chromosome X, males have only one. Also, one of the female X chromosomes may be inactivated. Therefore, special test statistics and quality control procedures are required. Thus, little is known about the role of X-chromosomal variants in CAD. To fill this gap, we conducted a comprehensive X-chromosome-wide meta-analysis including more than 43,000 CAD cases and 58,000 controls from 35 international study cohorts. For quality control, sex-specific filters were used to adequately take the special structure of X-chromosomal data into account. For single study analyses, several logistic regression models were calculated allowing for inactivation of one female X-chromosome, adjusting for sex and investigating interactions between sex and genetic variants. Then, meta-analyses including all 35 studies were conducted using random effects models. None of the investigated models revealed genome-wide significant associations for any variant. Although we analyzed the largest-to-date sample, currently available methods were not able to detect any associations of X-chromosomal variants with CAD.

In the last years, genome-wide association studies (GWAS) have uncovered numerous chromosomal risk loci for various complex diseases. Specifically, for coronary artery disease (CAD), 58 independent risk loci have been identified and verified in independent replication datasets^1^. However, a large part of the estimated heritability of CAD is not yet explained. This could be due partly to the fact that the X chromosome has routinely been excluded from GWAS. One reason for this is that the data has a different, sex-specific structure and, therefore, requires special analytical tools including special quality control and test statistics^2^. Thus, despite the profound effects of gender on the manifestation of CAD, no systematic association analyses of X-chromosomal variants with CAD have been reported so far. Therefore, an analysis of the X-chromosome from GWAS data might help to narrow the gap of missing heritability and help to yield new insights into the genetics of CAD.

X-chromosomal variants might be expected to play a role in the pathophysiology, since sex-specific features are known for CAD. Specifically, the risk to develop CAD varies between males and females independent from other risk factors. The symptoms of myocardial infarction (MI) as well as the prognosis after MI differ between males and females. Males are more likely than females to manifest CAD at young age, but females are more likely than males to die of a first MI. Furthermore, heart disease is the most common cause of death for females^3^. Thus, the analysis of X-chromosomal variants could help to explain the sex differences in CAD.

To comprehensively investigate the association of variants on chromosome X and CAD, we collected data from 35 world-wide study cohorts. All participating studies were part of the CARDIoGRAM + C4D consortium^1^. At each study site, quality control on subject level was performed, data were imputed on the basis of the 1000 genomes reference panel, and X chromosome-adapted association tests were calculated. After this, data were analyzed centrally at the University of Lübeck, where further quality control and the meta-analysis of all 35 studies were conducted. In the following, we will present the results of the association analysis of about 200,000 X-chromosomal single nucleotide polymorphisms (SNPs) with CAD on a sample of more than 100,000 subjects including more than 43,000 cases and 58,000 controls.

## Results

Details on the investigated studies are summarized in Table 1. For each of the 35 studies, logistic regression models with additive scoring for the SNP were used. To account for the sex-specific structure of X-chromosomal data, sex was always included as a covariate. In addition, interactions between SNP and sex were investigated. Where appropriate, further covariates could be included. Since one of the two female X-chromosomes may or may not be inactivated at a specific locus, models were calculated that assumed inactivation as well as not assuming inactivation.

The study-wise numbers of SNPs excluded due to quality control are given in Supplementary Tables S1 and S2. The inspection of inflation factors^4^ and Q-Q-plots (see also Supplementary Fig. S1) did not reveal any systematic inflation of specific studies. Thus, all studies were included in the meta-analysis.

**Figure 1:**
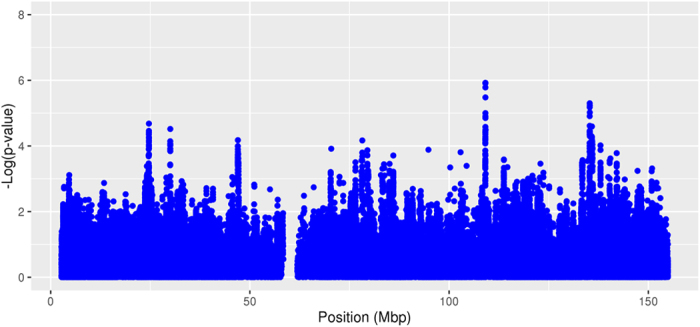
Chromosome-wide association results. The statistical model assumes no inactivation and no SNP*sex interaction. Shown are logarithmized random effects p-values of all 184,673 quality controlled SNPs in order of physical position in mega base pairs (mbp).

None of the statistical models used for the meta-analysis revealed a genome-wide significant association with CAD for any SNP. Association results for the model without inactivation assumption and without SNP*sex interaction are presented in Fig. 1. Results of the other models are comparable and presented in Supplementary Figs S2–S6.

### Subgroup and sensitivity analyses

To investigate possible sex-specific effects, we conducted subgroup analyses of males and females separately. Association plots of these models are presented in Supplementary Figs S7 and S8. Again, no genome-wide significant associations were to be observed. To exclude a possible bias introduced by including study cohorts of non-European ancestry, we performed a subgroup analysis including only the 31 studies with European background. Excluding non-European studies did not show additional associations either (Supplementary Figs S9–S14). Finally, to eliminate possible influences of the quality control parameters, we varied our criteria on missing frequencies and imputation quality. Performing stricter quality control reduced the number of SNPs available for meta-analysis to about 90,000 (depending on model, between 90,658 and 96,502). Results of these analyses were comparable to the results from the primary analyses. Thus, none of the sensitivity analyses did reveal novel associations, supporting the null findings of the main analyses.

### Power

**Figure 2:**
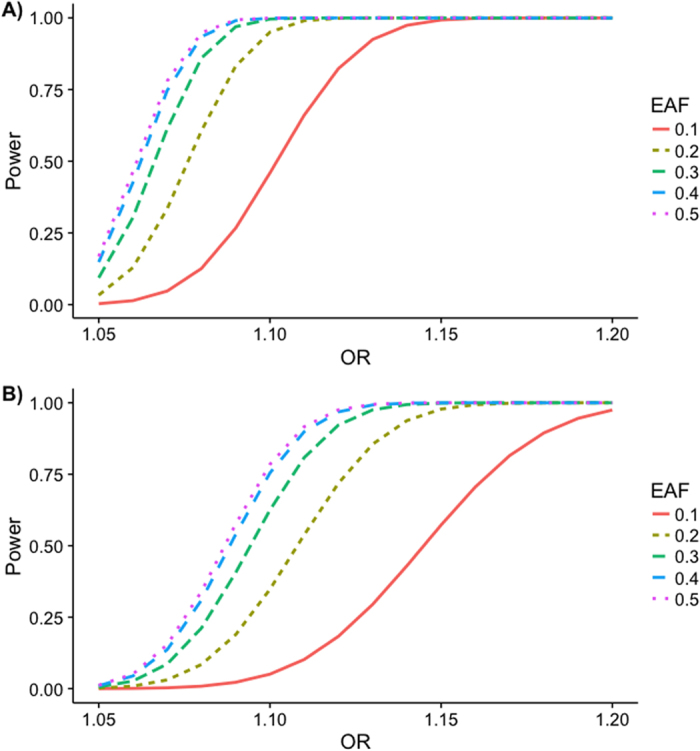
Estimated power. The power to detect an effect was estimated in dependence of the odds ratio (OR) and the effect allele frequency (EAF) using software Quanto (version 1.2.4 from May 2009). Parameters used for simulation: Binary (disease) phenotype, significance level α = 5·10^−8^, disease prevalence k_P_ = 0.1, log-additive genetic model, no gene-environment interaction. (**A**) Effective N_cases_ = 27,640, 1.5817 effective controls per effective case (corresponding to 43,718 effective controls), (**B**) Female N_cases_ = 12,160, 2.3968 female controls per female case (corresponding to 29,145 female controls).

We estimated the power of our study overall as well as in the smallest subgroup analyzed, i.e. the subgroup of females, as functions of the odds ratio and the effect allele frequency (EAF) (Fig. 2). In the entire sample of males and females, odds ratios of 1.1 or 1.11 would have reliably been detected with an EAF of 0.1 or higher. Even in the female subgroup, odds ratios of 1.15 and higher would have been detectable with a sufficiently large probability.

## Discussion

Although the presented meta-analyses included more than 100,000 subjects (the largest to date), with 43,120 CAD cases (28.2% women) and 58,291 controls (50% women), no genome-wide significant associations could be detected. This negative finding was independent of the model chosen for analysis. There were no sex-specific associations for CAD. Stricter quality control or excluding non-European studies did not reveal any different findings.

The NHGRI GWAS catalog^5^,^6^ reports 52 genome-wide significant associations of variants on chromosome X with more than 600 traits. All of the reported studies are of much smaller sample size (less than 50,000 samples) and used fewer methods than our analyses. Yet, they successfully discovered associations. However, no genome-wide significant associations with CAD or any other correlated phenotype have been reported on the X chromosome. The only gene reported for CAD on chromosome X is *CHRDL1* with a p-value of 9 · 10^−7^ for rs5943057^7^, but this did not replicate in our analyses (p = 0.0172 for the model without inactivation assumption and without SNP*sex interaction). As our power estimates indicated (Fig. 2), in such a large dataset, the statistical power to detect medium to large effects is high, and only small effects are likely to have been missed. Therefore, the most natural explanation to the negative finding of this meta-analysis is that there are no substantial associations of X-chromosomal variants with CAD.

However, since the progression and the symptoms of CAD, as well as the prognosis after MI, are sex-specific, it may be that the genetics of chromosome X are more complex than previously assumed. For example, the inactivation patterns are not yet understood completely, and it has been shown that inactivation of the female X-chromosome can be cell-specific. The silenced X-chromosome is not necessarily chosen randomly, and silenced regions can differ between females^8^,^9^. Although we evaluated models with and without the assumption of inactivation, different inactivation patterns between females at one locus were not taken into account. Nor was non-random inactivation incorporated into the model. This could affect the power of the test statistics that were used and might result in an analysis that is less powerful than estimated. Further, it might be argued that the use of other statistical methods could have yielded significant findings. Specifically, most other studies (e.g. refs 10 and 11) are based on a separate analysis of males and females with a subsequent summary by a classical or sex-specific^12^ meta-analytic method. In contrast, we followed the approach to directly compute joint test statistics for males and females, taking the different structure of chromosome X in the sexes into account. However, examples for both methodological approaches have been compared in simulations^13^,^14^ with the overall result that the joint tests have greater power unless there are relevant differences in the effect sizes between males and females, which is not to be expected given our sex-specific subgroup analyses. Another potential limitation could be the lower coverage of chromosome X than autosomal chromosomes^2^,^15^. Specifically, depending on the specific genotyping chip, the distance between two known variations^16^ averages roughly 700 to 10,000 bp on all chromosomes but about 1400 to 22,500 bp on chromosome X. Accordingly, the median distance in our studies is 11,300 bp, so that the coverage is less than optimal but comparable to the use of an older genotyping array in general. More generally, the use of these technologies is restricted to finding associations with SNPs only; the effect of other structural variants or even having XX instead of XY cannot thus be detected. Another explanation for the lack of significant associations could be problems with the imputation. Using the IMPUTE2 algorithm, as most of the study sites did, a mixture of two populations, males and females, is imputed together. Perhaps this leads to a bias that has not been taken adequately into account. From a clinical perspective, coronary disease and its manifestations differ in women, including a larger proportion of younger women with myocardial infarction having the distinct pathophysiology of coronary dissection, which could complicate the dissection of genetic influence according to sex.

Although we have analyzed the largest sample to date, we were not able to detect genome-wide significant associations between chromosome X variants and CAD with currently available methods. Due to this lack of significant associations, the sex-specific differences in CAD are still unexplained. The genetics of chromosome X may be more complex than has been assumed, so that more sophisticated test statistics which allow for these complex biological processes would be required to detect associations of variants on the X-chromosome with CAD.

## Materials and Methods

### Sex-specific structure of chromosome X

One reason why chromosome X is usually excluded from GWAS is that the data has a different, sex-specific structure and, therefore, requires special analytical tools^15^,^17^. While there are two copies of each autosomal chromosome, males carry only one copy of the X chromosome whereas females, again, carry two copies. Therefore, at each SNP, females can carry one of three possible genotypes; that is, they can have 0, 1 or 2 copies of a specific allele. In contrast to this, there are only two possible genotypes for males, corresponding to 0 or 1 copies of a specific allele. Only for the so-called pseudo-autosomal regions, there exist homologous loci on the Y chromosome, and males can have up to 2 copies of a specific allele. In addition, one of the two female X chromosomes might be inactivated. In each cell, one of the two female X chromosomes is randomly selected to be silenced^18^. This means that the expression levels of this chromosome are much lower than for the second chromosome in the cell. This mechanism of dose compensation should result in comparable expression levels for males and females despite the different number of chromosome copies. However, this inactivation is incomplete: while some genes or regions will be completely inactivated, some genes might show expression levels that are reduced only slightly or not at all^8^. Therefore, to analyze X-chromosomal data, special quality control and test statistics are required^2^. Most of the quality control needs to be done separately for males and females, and test statistics for chromosome X should take into account the different data structure for males and females, for example by including sex as a covariate into the model. The choice of the best statistical test depends on the underlying genetic model and the inactivation patterns at a specific locus^13^.

### Study cohorts

The meta-analysis includes data from 43,120 cases with CAD and 58,291 controls from 35 studies with 28.2% female cases and 50.0% female controls (for details, see Table 1). A subject was regarded as a CAD case if he/she had an inclusive CAD diagnosis, e.g. MI, acute coronary syndrome, chronic stable angina, or coronary stenosis >50%. More detailed information on the study cohorts can be found in the meta-analysis of autosomal variants of the CARDIoGRAMplusC4D Consortium^1^ which included most of the samples presented here. Thirty-one of the 35 studies consist of subjects with European ancestry, two study cohorts are of Asian ancestry, one of Hispanic and one of African ancestry.

### Genotyping and imputation to 1000G data

Details on the genotyping arrays used for each study cohort have been published before^1^. At each study site, untyped SNPs were imputed on the basis of the 1000 genomes phase 1 version 3 reference panel^19^. Although this reference panel includes insertion and deletion variants (indels), these were excluded from further analyses. Prior to any quality control, there were 1,193,934 SNPs available for the non-pseudo-autosomal region of chromosome X.

### Quality control

Quality control at subject level and at variant level was performed at each study site prior to imputation and association analysis as described previously^1^. In addition to quality criteria typically used for analyses of autosomal SNPs^20^,^21^, all subjects for whom the genotypic and reported sex could not be assigned unambiguously were excluded. Post-imputation quality control at SNP level was done centrally and in the same manner for all contributing studies. Here, SNPs were excluded if one of the following criteria was fulfilled: (1) ≥25% missing genotypes in either female or male cases or controls, (2) deviation from Hardy-Weinberg equilibrium in female controls with p < 0.0001, (3) minor allele frequency <1% in either males or females, and (4) imputation quality score (INFO for IMPUTE2^22^,^23^,^24^,^25^ and r^2^ for Minimac^22^,^26^) <0.5. For sensitivity analysis, we additionally applied stricter criteria for imputation quality (INFO >0.7) and missing genotypes (<2% in either female or male cases or controls).

### Study-wise association analysis

Study-wise association analyses were calculated at each study site. Logistic regression models with additive scoring for the SNP were used. The sex-specific structure of X-chromosomal data implies different variances in male and female sub-samples. To account for this, sex needs to be included as a covariate in the model. In addition, interactions between SNP and sex were investigated. Where appropriate, additional covariates to adjust for population stratification have been included in the model, for example in the form of variables calculated from a principal component analysis or variables describing the ethnic background of the subjects.

Since one of the two female X-chromosomes may or may not be inactivated at a specific locus, models were calculated that assumed inactivation as well as not assuming inactivation. If inactivation is present at a locus, two risk alleles of a female subject should show similar expression levels as compared to one risk allele in a male individual. Therefore, while the female genotypes for such a SNP are coded 0, 1 or 2, according to 0, 1 or 2 alleles, the genotypes for males should be coded 0 or 2 according to 0 or 1 alleles. If no inactivation occurs, the expression levels of one allele in females should be the same as one allele in males. Therefore, while the coding of female genotypes is unchanged, male genotypes should now be coded 0 or 1, according to 0 or 1 alleles. As an alternative to assuming complete or no inactivation approach, Wang *et al*.^27^ proposed likelihood ratio tests for the situation of non-random or skewed inactivation, which can be more powerful in the case of non-random inactivation. However, given that the gain in power is small and that these tests are available in Matlab^28^ only, which was not available for many of the participating study sites, we refrained from using this particular approach. These considerations resulted in four models being investigated (Table 2).

### Post-hoc quality control

After calculation of the association analyses for each single study, post-hoc quality control was applied for all studies. To control for population stratification or other sources of inflation of p-values, inflation factors according to Devlin and Roeder^4^ were calculated, and plots of expected versus observed test statistics were inspected visually. In addition, for each SNP, mean EAFs over all studies were calculated and compared to the study-wise EAFs. SNPs with extreme deviations (>0.1, corresponding to more than 4 standard deviations) from the mean EAF were excluded from further analyses. Finally, only SNPs for which at least half of the studies were available were included in the meta-analyses.

### Meta-analyses

For the meta-analyses of the 35 studies, random effect models were calculated for each of the four models defined in Table 2. In the same way, meta-analyses of the effect estimates for the SNP*sex interaction were performed. In all of these analyses, outlier analyses according to Preuß *et al*.^20^ were performed to exclude studies with extremely inflated effect estimates.

### Sensitivity and subgroup analyses

We conducted the following sensitivity and subgroup analyses: (1) Subgroup analyses of males and females separately with subsequent meta-analyses to gain further insight into sex-specific effects; (2) Subgroup meta-analysis including only the 31 studies with European background; (3) Meta-analysis of SNPs fulfilling stricter quality criteria as described above.

### Power estimation

Using the software Quanto, version 1.2.4^29^, we estimated the power of our analyses in two ways. Firstly, to take into account our entire sample of males and females, a simple combination of the data is not possible due to the sex-specific structure of X chromosomal variants. We therefore followed Clayton^30^ in assuming that the variance in males has twice the size of that in females in the additive model assuming inactivation. Therefore, we assumed that the effective sample size in males is halved, and this was added to the female sample size. Power was then estimated as a function of the odds ratio and the EAF at a significance level of α = 5·10^−8^, a disease prevalence k_P_ = 0.1, and a log-additive genetic model. Secondly, we estimated the power for the smallest subgroup analyzed, i.e. the subgroup of females. The parameters in Quanto were set to the same values as before.

## Additional Information

**How to cite this article**: Loley, C. *et al*. No Association of Coronary Artery Disease with X-Chromosomal Variants in Comprehensive International Meta-Analysis. *Sci. Rep*. **6**, 35278; doi: 10.1038/srep35278 (2016).

## Supplementary Material

Supplementary Information

## Figures and Tables

**Table 1:** Cohort descriptives of the 35 studies participating in the 1000G coronary artery disease meta-analysis of the X-chromosome.

Study	Ancestry	Cases (% females)	Controls (% females)	% MI* (in cases)
ADVANCE	White European	275 (59.9)	311 (60.7)	100.0
BioMe_AfrAm	African American	362 (66.5)	2778 (70.9)	36.0
BioMe_EurAm	European American	487 (35.0)	1382 (61.6)	30.4
BioMe_HisAm	Hispanic American	758 (55.1)	3338 (71.3)	36.7
Cardiogenics	White European	391 (13.7)	410 (60.8)	12.5
CATHGEN	White European	1191 (31.4)	646 (58.9)	48.1
CCGB_2	White European	1547 (21.8)	344 (45.0)	60.3
EGCUT	White European	658 (49.6)	5841 (56.1)	19.6
FGENTCARD	Libanese	1802 (25.0)	466 (50.8)	16.0
FINCAVAS	Finnish European	774 (21.2)	647 (44.8)	12.4
FINRISK/PredictCVD	Finnish European	677 (30.9)	1200 (42.7)	40.0
GerMIFSI	White European	637 (33.9)	1644 (51.2)	100.0
GerMIFSII	White European	1222 (21.0)	1298 (48.5)	100.0
GerMIFSIII	White European	1096 (20.5)	1509 (52.2)	100.0
GerMIFSIV	White European	1002 (36.0)	1147 (61.9)	100.0
GerMIFSV	White European	2459 (24.5)	1611 (53.0)	100.0
HPS	White European	2700 (23.9)	2748 (72.5)	65.0
HSDS	White European	206 (0.0)	258 (0.0)	45.6
ITH	White European	402 (29.4)	449 (32.9)	100.0
LIFE-Heart	White European	1531 (24.8)	768 (50.3)	44.0
LOLIPOP	Indian Asian	2791 (18.5)	3757 (14.1)	43.9
LURIC	White European	2347 (25.5)	621 (48.0)	62.9
MEDSTAR	White European	875 (34.4)	447 (55.9)	100.0
MIGEN	White European	2905 (24.8)	2998 (26.9)	100.0
OHGS_A2	White European	921 (24.9)	1001 (50.5)	64.3
OHGS_B2	White European	1183 (22.4)	1391 (49.5)	55.6
OHGS_C2	White European	833 (6.4)	317 (66.7)	44.3
PENNCATH	White European	933 (29.2)	468 (58.8)	100.0
PIVUS	White European	119 (24.3)	830 (54.7)	77.3
PROCARDIS	White European	5719 (29.2)	6526 (62.5)	80.0
SDS	Indian Asian	176 (29.5)	1421 (49.0)	100.0
THISEAS	White European	423 (21.3)	593 (56.3)	60.1
TWINGENE	White European	814 (29.1)	5999 (55.8)	70.5
ULSAM	White European	322 (0.0)	857 (0.0)	84.2
WTCCC	White European	1922 (20.9)	2930 (51.2)	71.5
**Total**	—	**43120 (28.2)**	**58291 (50.0)**	**66.7**

^*^MI = myocardial infarction.

**Table 2:** Association models for chromosome X.

Model	Mathematical description^a^	SNP*sex interaction	Coding of SNP	Inactivation
I	*Logit*(CAD)* = β*_*0*_* + β*_*1*_ · SNP* + β*_*2*_ · sex	No	Females: 0, 1, or 2; Males: 0 or 1	No
II	*Logit*(CAD)* = β*_*0*_* + β*_*1*_ · SNP* + β*_*2*_ · sex	No	Females: 0, 1, or 2; Males: 0 or 2	Yes
III	*Logit*(CAD)* = β*_*0*_* + β*_*1*_ · SNP* + β*_*2*_ · sex* + β*_*3*_ · SNP*sex	Yes	Females: 0, 1, or 2; Males: 0 or 1	No
IV	*Logit*(CAD)* = β*_*0*_* + β*_*1*_ · SNP* + β*_*2*_ · sex* + β*_*3*_ · SNP*sex	Yes	Females: 0, 1, or 2; Males: 0 or 2	Yes

^a^For each study, further covariates to adjust for population stratification have been added to the model where appropriate.
