# Supplementary material for: No Association of Coronary Artery Disease with X-Chromosomal Variants in Comprehensive International Meta-Analysis
Source: Sci Rep. 2016 Oct 12;6:35278. doi: 10.1038/srep35278 (PMC5059659; doi:10.1038/srep35278)
Supplement: Supplementary Information [file srep35278-s1.pdf]

Supporting Information on "No Association of Coronary Artery Disease with X-Chromosomal Variants in Comprehensive International Meta-Analysis"

Christina Loley[1,2], Maris Alver[3,4], Themistocles L. Assimes[5], Andrew Bjonnes[6], Anuj Goel[7,8], Stefan Gustafsson[9], Jussi Hernesniemi[10,11], Jemma C. Hopewell[12], Stavroula Kanoni[13], Marcus E. Kleber[14], King Wai Lau[12], Yingchang Lu[15], Leo-Pekka Lyytikäinen[10,16], Christopher P. Nelson[17,18], Majid Nikpay[19], Liming Qu[20], Elias Salfati[5], Markus Scholz[21,22], Taru Tukiainen[23,24], Christina Willenborg[2,25], Hong-Hee Won[26], Lingyao Zeng[27,28], Weihua Zhang[29,30], Sonia S. Anand[31], Frank Beutner[22,32], Erwin P Bottinger[15], Robert Clarke[12], George Dedoussis[33], Ron Do[15,34,35,36], Tõnu Esko[3,37], Markku Eskola[11], Martin Farrall[7,8], Dominique Gauguier[38], Vilmantas Giedraitis[39], Christopher B. Granger[40], Alistair S. Hall[41], Anders Hamsten[42], Stanley L. Hazen[43], Jie Huang[44], Mika Kähönen[45,46], Theodosios Kyriakou[7,8], Prof. Reijo Laaksonen[10,16,47], Lars Lind[48], Cecilia Lindgren[8,49], Patrik K. E. Magnusson[50], Eirini Marouli[13], Evelin Mihailov[3], Andrew P. Morris[8,51], Kjell Nikus[11], Nancy Pedersen[50], Loukianos Rallidis[52], Veikko Salomaa[53], Svati H. Shah[40], Alexandre F. R. Stewart[19], John R. Thompson[54], Pierre A. Zalloua[55,56], John C. Chambers[30,31,57], Rory Collins[12], Erik Ingelsson[8,9], Carlos Iribarren[58], Pekka J. Karhunen[10,59], Jaspal S. Kooner[31,57,60], Terho Lehtimäki[10,16], Ruth J. F. Loos[15,61], Winfried März[14,62,63], Ruth McPherson[19], Andres Metspalu[3,4], Muredach P. Reilly[64], Samuli Ripatti[58,65,66], Dharambir K. Sanghera[67,68,69], Joachim Thiery[22,70], Hugh Watkins[7,8], Panos Deloukas[13,71,72], Sekar Kathiresan[6,24,37,73], Nilesh J. Samani[17,18], Heribert Schunkert[28,29], Jeanette Erdmann[2,25,*], Inke R. König[1,2,*]

Supplementary Tables

Table S1. Numbers of SNP after quality control and inflation factors for genotyped SNPs for each model.

Study	SNPs after QC		Inflation factors					
	All	Genotyped	I	II	III	IV	III, interaction	IV, interaction
ADVANCE	177,691	11,899	1.09	1.01	0.98	0.98	0.95	0.97
Cardiogenics	192,021	11,890	1.07	1.05	0.99	0.99	0.97	0.97
CCGB	176,848	22,923	1.02	1.06	1.16	1.16	1.04	1.03
DUKE	193,078	13,741	0.97	0.98	0.98	0.99	0.99	0.97
EGCUT	210,121	13,927	0.89	0.94	0.92	0.92	0.94	0.94
FGENTCARD	86,148	4,473	1.03	1.09	1.06	1.06	1.00	0.96
FINCAVAS	2,257	91	0.73	0.82	1.02	1.02	1.51	1.02
GerMIFSI	141,599	6,373	1.20	1.30	1.24	1.24	1.06	0.99
GerMIFSII	186,729	22,029	1.09	1.06	1.03	1.03	1.03	1.04
GerMIFSIII	158,017	6,953	1.07	0.99	0.98	0.93	0.93	0.93
GerMIFSIV	186,872	23,405	1.11	1.03	1.03	1.03	1.05	1.07

GerMIFSV	204,083	12,114	1.04	1.06	1.08	1.08	0.98	0.98
HPS	156,612	9,792	1.03	1.03	1.04	1.04	1.07	1.09
HSDS	255,101	25,973	0.98	0.98	NA	NA	NA	NA
BioMe-AfrAm	338,799	7,944	1.00	0.97	1.00	1.00	1.00	1.01
BioMe-EurAm	187,593	7,277	1.02	1.01	0.99	0.99	1.01	1.05
BioMe-HisAm	286,975	7,936	0.99	1.03	1.15	1.15	1.17	1.13
ITH	181,195	23,784	0.96	0.99	1.05	1.05	1.00	0.97
LIFE	210,461	13,323	0.99	0.97	1.02	1.02	1.03	1.06
LOLIPOP	178,438	13,382	1.04	1.09	1.12	1.12	1.03	1.01
LURIC	187,338	23,567	0.97	0.98	1.00	1.00	1.02	1.00
MEDSTAR	206,446	26,957	1.02	1.01	0.99	0.99	1.06	1.09
MIGEN	231,686	26,661	1.09	1.14	1.16	1.16	1.04	1.09
OHGS_A	86,940	4,357	1.11	1.08	0.95	0.95	0.91	0.91
OHGS_B	178,104	23,072	1.23	1.22	1.10	1.10	1.09	1.09
OHGS_C	191,035	14,186	1.02	1.02	0.97	0.97	0.99	1.01
PENNCATH	204,454	26,940	0.95	0.97	0.98	0.98	0.93	0.94
PIVUS	168,123	14,497	1.02	1.00	0.95	0.95	0.99	0.98

PredictCVD	220,913	14,716	1.06	1.04	1.06	1.06	1.03	1.02
PROCARDIS	187,691	10,383	1.04	1.03	1.37	1.07	1.04	1.06
SDS	218,982	11,067	1.03	1.08	1.07	1.07	0.99	0.96
THISEAS	197,562	14,819	1.01	1.11	1.15	1.15	1.05	1.02
TWINGENE	149,289	14,472	0.99	0.93	0.92	0.92	0.98	1.00
ULSAM	213,695	31,466	1.04	1.04	NA	NA	NA	NA
WTCCC	147,698	7,656	1.16	1.17	1.07	1.07	1.04	1.02

QC: Quality control. I, model assuming no inactivation, no SNP*sex interaction. II, model assuming inactivation, no SNP*sex interaction. III, model assuming no inactivation, but SNP*sex interaction, inflation factors for SNP. IV, model assuming inactivation and SNP*sex interaction, inflation factors for SNP. III, interaction, model assuming no inactivation, but SNP*sex interaction, inflation factors for SNP*sex interaction. IV, interaction, model assuming inactivation and SNP*sex interaction, inflation factors for SNP*sex interaction.

Table S2. Number of SNP excluded because study-wise effect allele frequency (EAF) differed more than 0.1 from mean EAF estimated over all studies.

Study	All SNPs	Excluded
ADVANCE	177,691	611
Cardiogenics	192,021	188
CCGB_2	176,848	18
DUKE_2	193,078	89
EGCUT	210,121	1,978
FGENTCARD	104,315	9,783
FINCAVAS	2,058	77
GerMIFSI	141,599	34
GerMIFSII	186,729	32
GerMIFSIII	158,017	5
GerMIFSIV	186,872	8
GerMIFSV	204,080	26
HPS	162,842	375
HSDS	230,705	6,471
BioMe_AfrAm	338,799	98,429

BioMe_EurAm	187,593	673
BioMe_HisAm	286,975	47,126
ITH	181,195	171
LIFE	210,461	74
LOLIPOP	178,311	47,664
LURIC	187,337	2
MEDSTAR	206,446	666
MIGEN	209,720	396
OHGS_A2	86,940	3,244
OHGS_B2	178,104	39
OHGS_C2	191,035	150
PENNCATH	204,454	696
PIVUS	168,118	817
PredictCVD	220,907	7,701
PROCARDIS	187,691	282
SDS	218,982	53,715
THISEAS	197,562	5,480

TWINGENE	149,289	182
ULSAM	213,694	202
WTCCC	147,698	22

Supplementary Figures

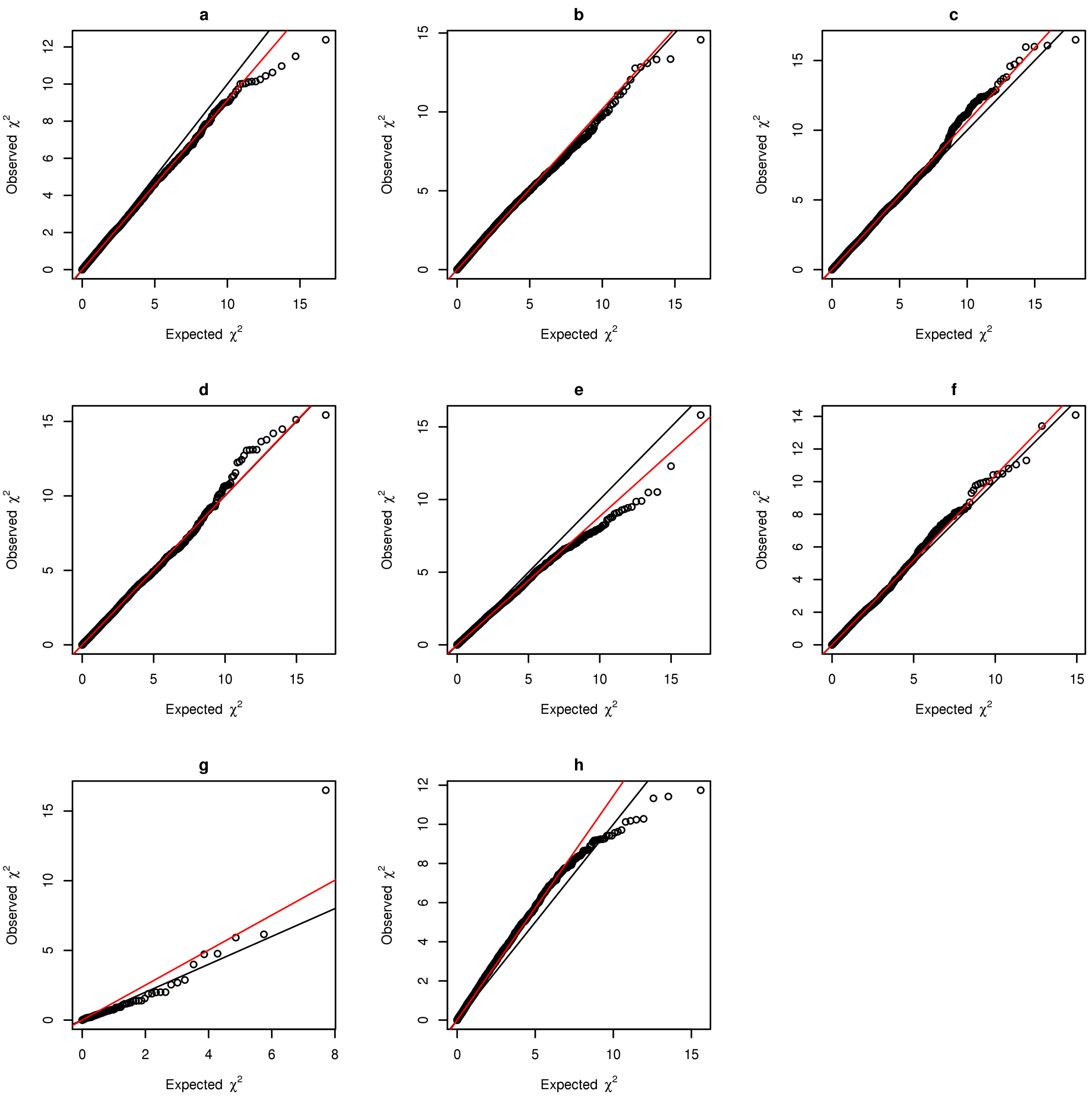

Figure S1. Q-Q plots of models and studies with genomic inflation factor λ > 1.2. Displayed are observed versus expected χ^2 test statistics of all genotyped SNPs. Red line, regression line. Black line, bisectrix / ideal regression line without inflation. **a**, FINCAVAS, model without inactivation assumption, but with SNP*sex interaction. **b**, GerMIFSI, model without inactivation assumption and without SNP*sex interaction. **c**, GerMIFSI, model with assumption of inactivation, but without SNP*sex interaction. **d**, GerMIFSI, model without

inactivation assumption, but with SNP*sex interaction. **e**, GerMIFSI, model with assumption of inactivation and with SNP*sex interaction. **f**, OHGS_B2, without inactivation assumption and without SNP*sex interaction. **g**, OHGS_B2, model with assumption of inactivation, but without SNP*sex interaction. **h**, PROCARDIS, model without inactivation assumption, but with SNP*sex interaction.

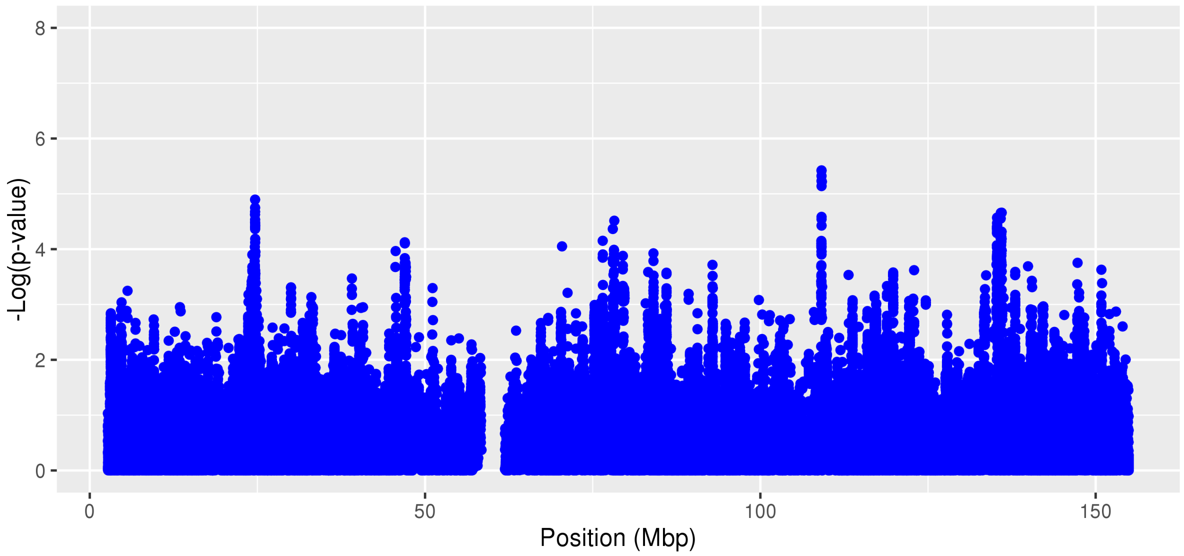

Figure S2. Association results of model with assumption of inactivation, but without SNP*sex interaction. Shown are logarithmized random effects p-values of all 184,682 quality controlled SNPs in order of physical position in mega base pairs (mbp).

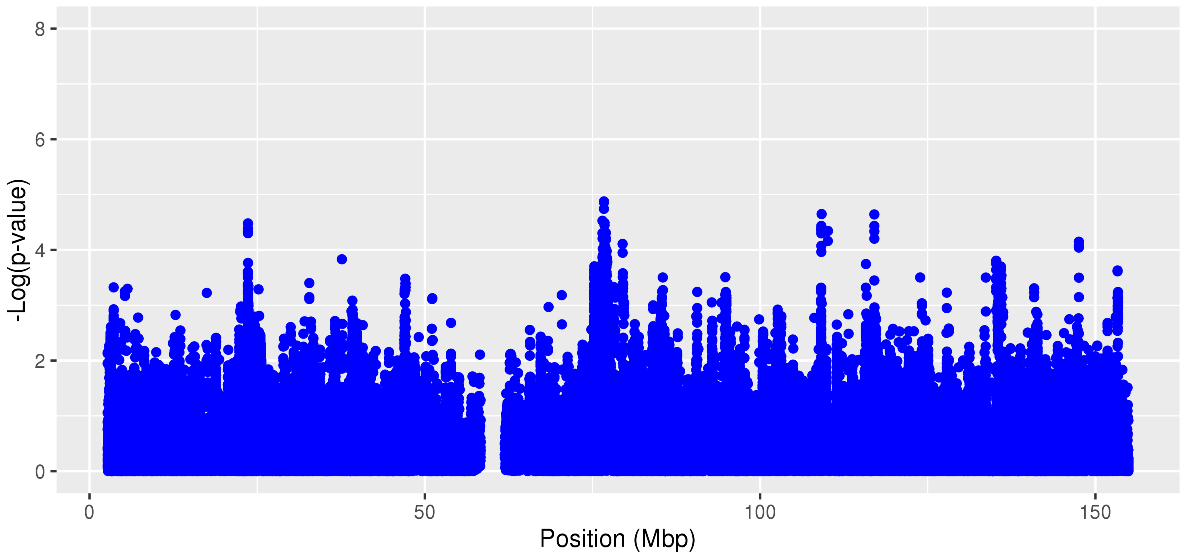

Figure S3. Association results of model without inactivation assumption, but with SNP*sex interaction. Shown are logarithmized random effects p-values for the SNP effect of all 183,058 quality controlled SNPs in order of physical position in mega base pairs (mbp).

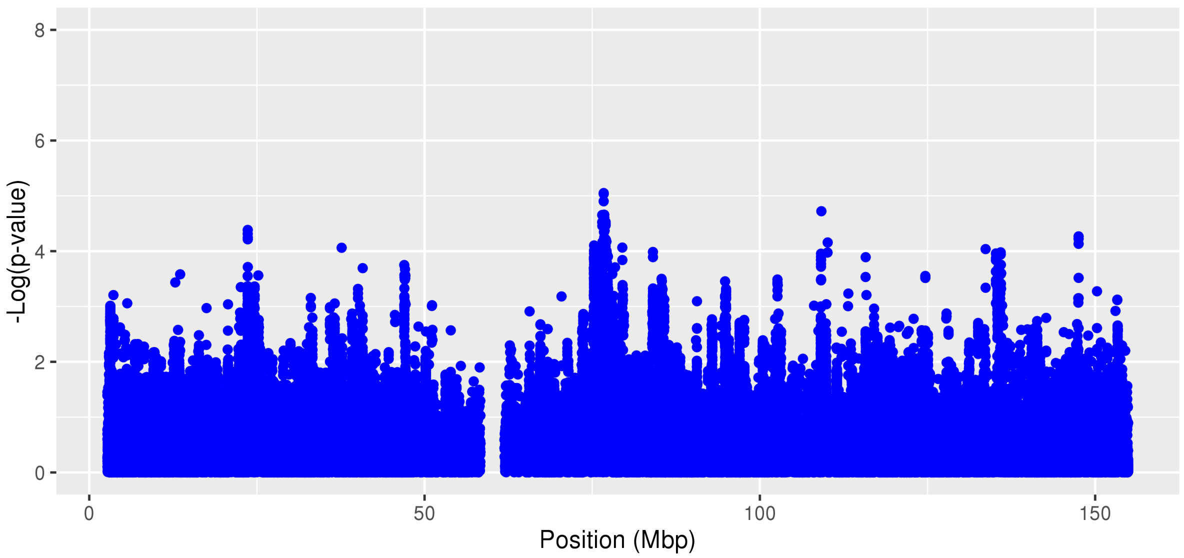

Figure S4. Association results of model with assumption of inactivation and with SNP*sex interaction. Shown are logarithmized random effects p-values for the SNP effect of all 183,059 quality controlled SNPs in order of physical position in mega base pairs (mbp).

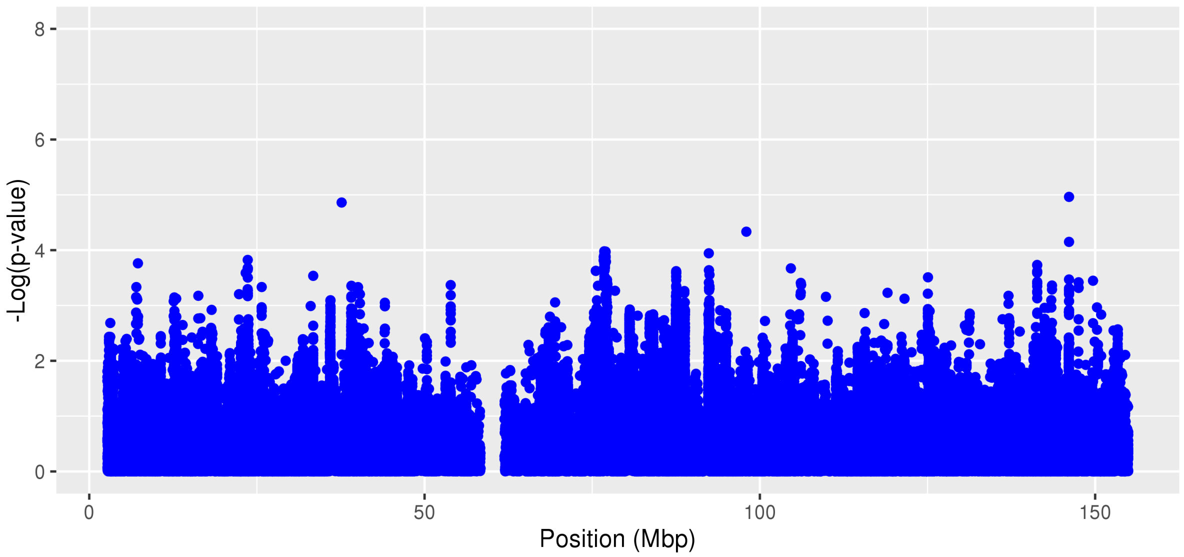

Figure S5. Interaction results of model without inactivation assumption, but with SNP*sex interaction. Shown are logarithmized random effects p-values for the SNP*sex interaction of all 183,055 quality controlled SNPs in order of physical position in mega base pairs (mbp).

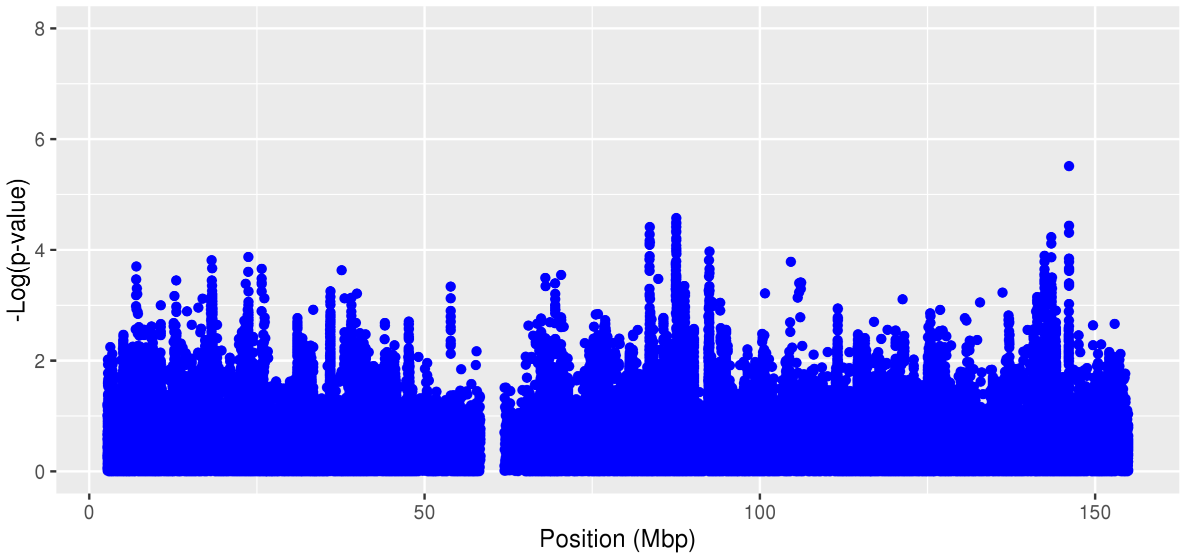

Figure S6. Interaction results of model with assumption of inactivation and with SNP*sex interaction. Shown are logarithmized random effects p-values for the SNP*sex

interaction of all 183,057 quality controlled SNPs in order of physical position in mega base pairs (mbp).

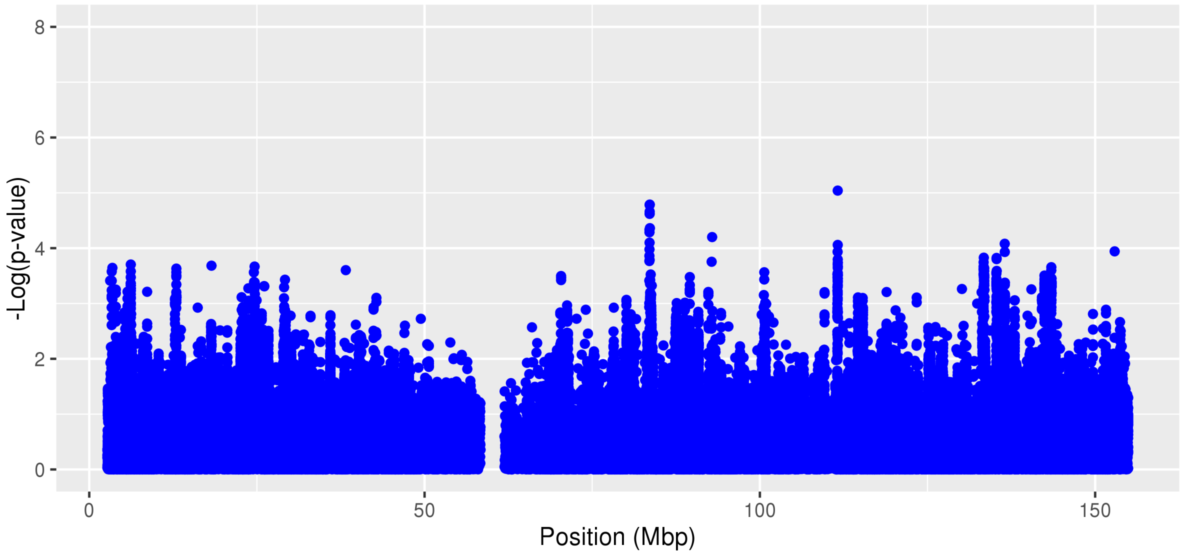

Figure S7. Association results for the female subgroup. Shown are logarithmized random effects p-values of all 181,776 quality controlled SNPs in order of physical position in mega base pairs (mbp).

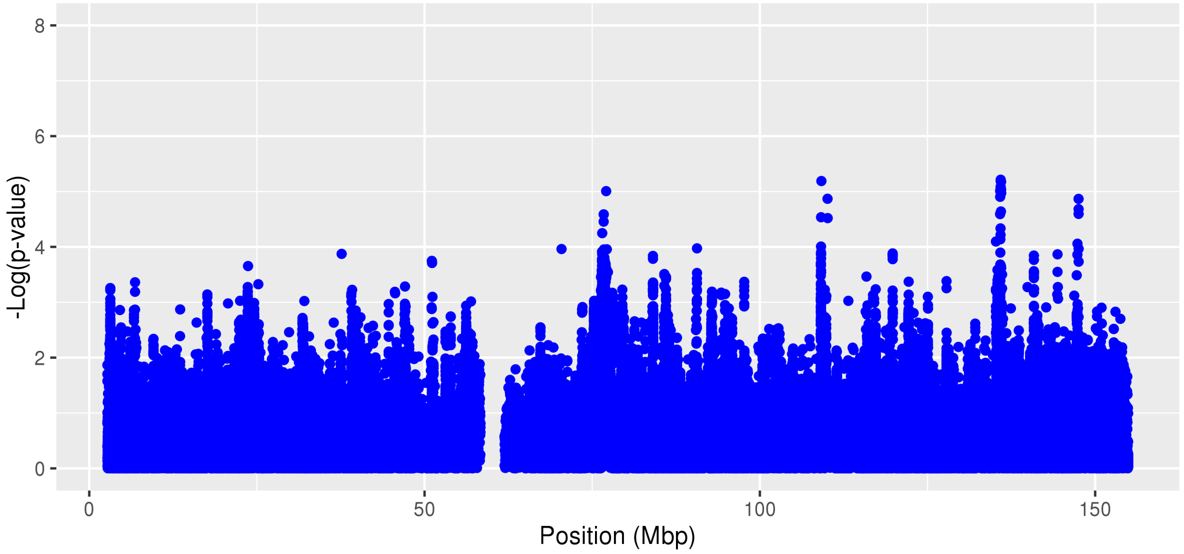

Figure S8. Association results for the male subgroup. Shown are logarithmized random effects p-values of all 181,854 quality controlled SNPs in order of physical position in mega base pairs (mbp).

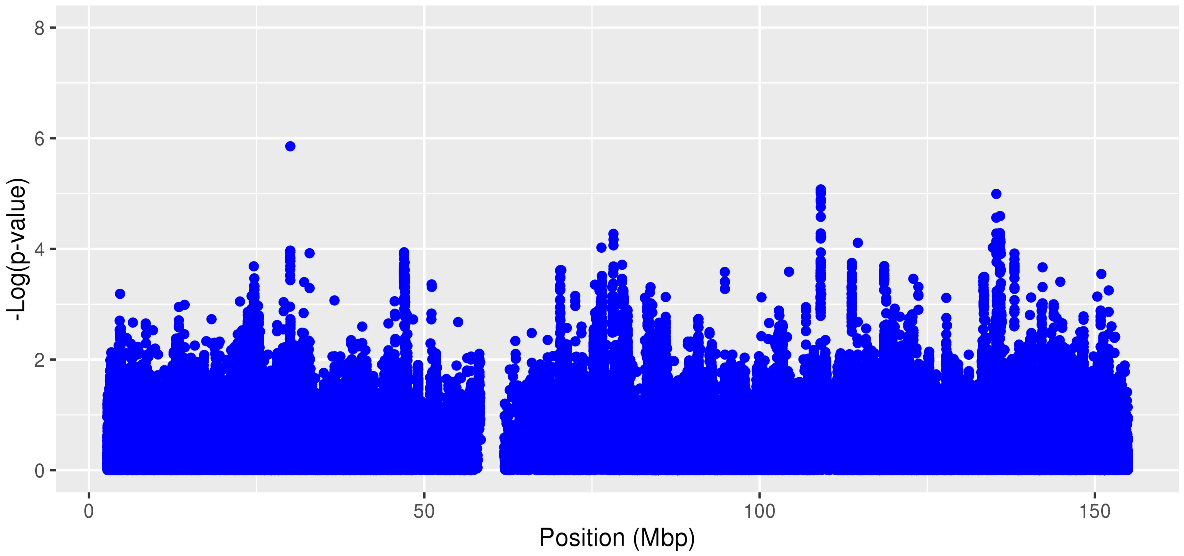

Figure S9. Association results for European studies, model without inactivation assumption and without SNP*sex interaction. Shown are logarithmized random effects p-values of all 186,564 quality controlled SNPs in order of physical position in mega base pairs (mbp) in order of physical position in mega base pairs (mbp).

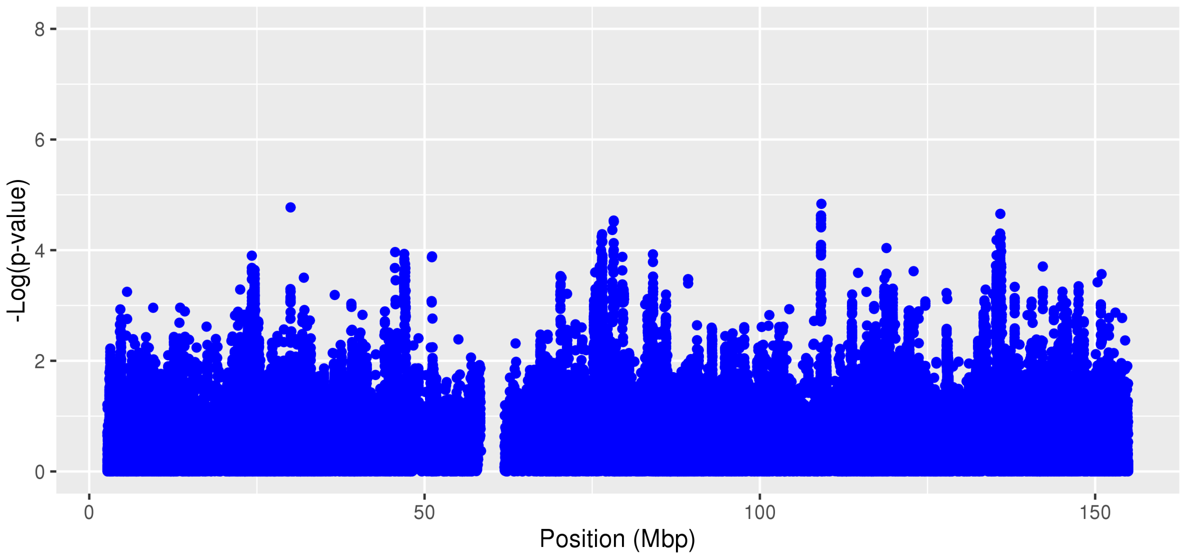

Figure S10. Association results for European studies, model with assumption of inactivation, but without SNP*sex interaction. Shown are logarithmized random effects p-values of all 186,583 quality controlled SNPs in order of physical position in mega base pairs (mbp).

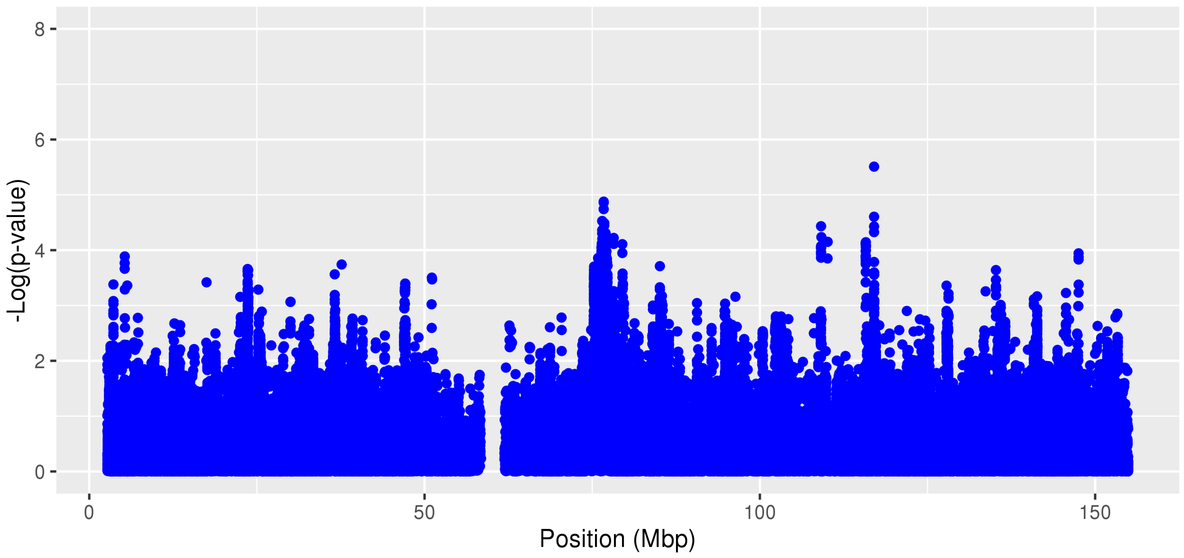

Figure S11. Association results for European studies, model without inactivation assumption, but with SNP*sex interaction. Shown are logarithmized random effects p-values for the SNP effect of all 185,046 quality controlled SNPs in order of physical position in mega base pairs (mbp).

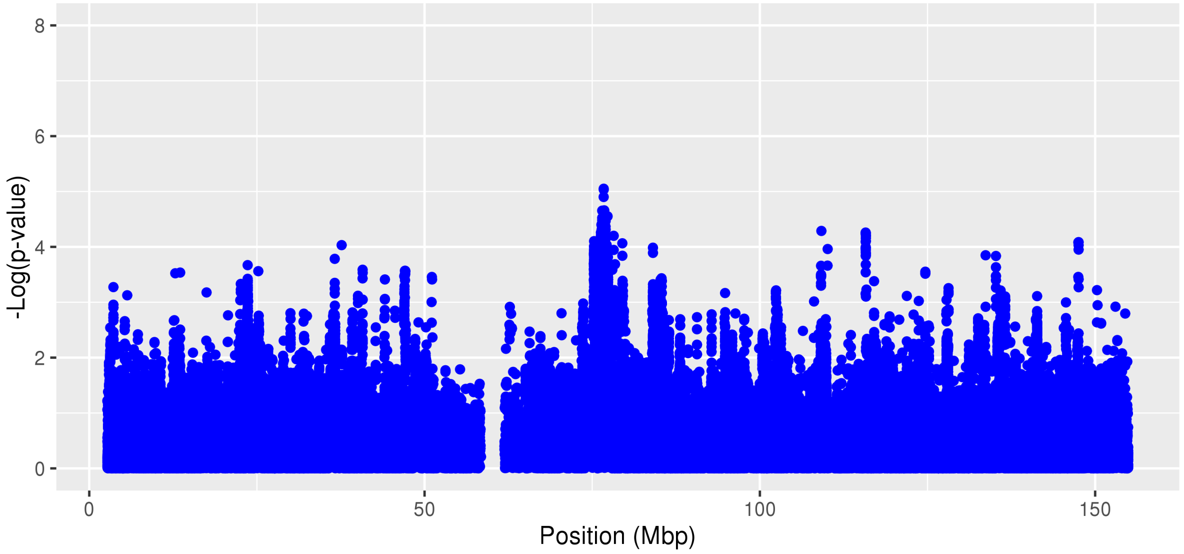

Figure S12. Association results for European studies, model with assumption of inactivation and with SNP*sex interaction. Shown are logarithmized random effects p-values for the SNP effect of all 185,041 quality controlled SNPs in order of physical position in mega base pairs (mbp).

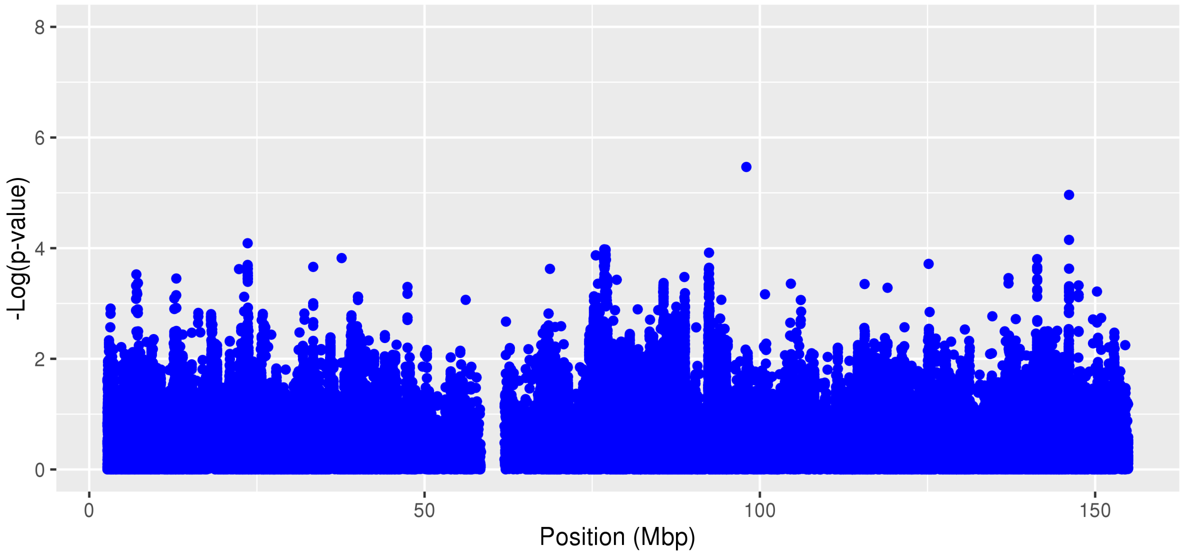

Figure S13. Interaction results for European studies, model without inactivation assumption, but with SNP*sex interaction. Shown are logarithmized random effects p-

values for the SNP*sex interaction of all 185,050 quality controlled SNPs in order of physical position in mega base pairs (mbp).

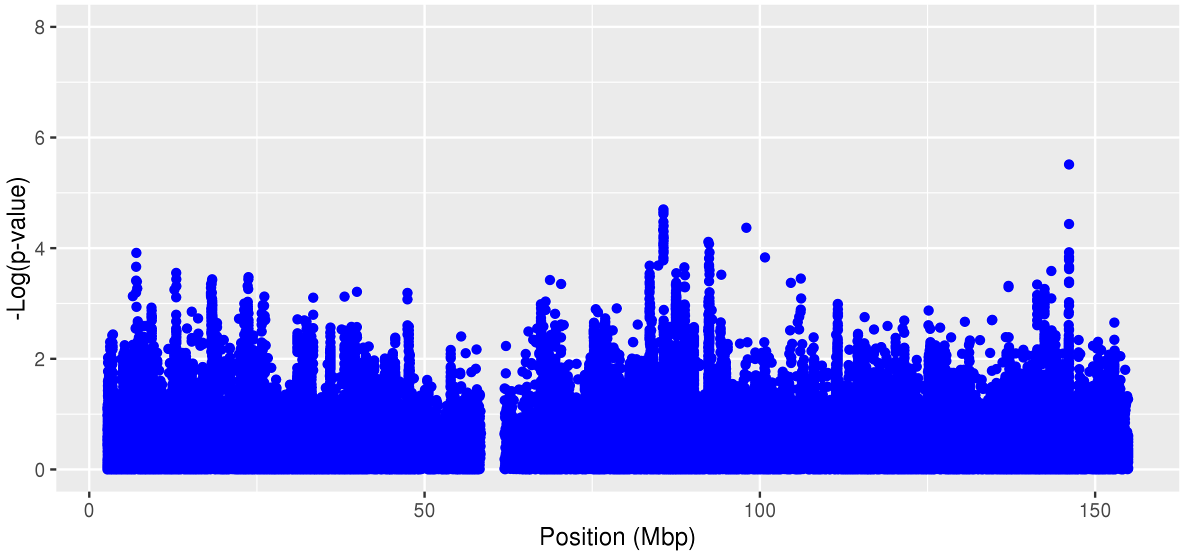

Figure S14. Interaction results for European studies, model with assumption of inactivation and with SNP*sex interaction. Shown are logarithmized random effects p-values for the SNP*sex interaction of all 185,051 quality controlled SNPs in order of physical position in mega base pairs (mbp).
